# The Load Structure in International Competitive Climbing

**DOI:** 10.3389/fspor.2022.790336

**Published:** 2022-03-22

**Authors:** Marvin Winkler, Stefan Künzell, Claudia Augste

**Affiliations:** Institute of Sports Science, Augsburg University, Augsburg, Germany

**Keywords:** competition analysis, elite climbing, bouldering, lead climbing, speed climbing, world cup

## Abstract

The analysis of the load structure in competitions is essential to develop performance structure models from which sport-specific testing and training protocols can be derived. The aim of this study was to characterize the external load structure of competitive climbing at an international level in the disciplines of speed, bouldering, lead, and Olympic combined based on video recordings of top athletes. In speed, the route was completed by women with a median of 11 moves and by men with 9 moves that required 0.73 and 0.60 s per move, respectively. Bouldering competitions are characterized by various bouts of activity with resting periods in between. Athletes attempted a boulder problem, a median of 3 times in the qualification and semi-final rounds and 4 times in the final round with an average attempt duration of 27.0 s. In lead, the load structure is characterized by an average climbing time of 4:09 min and 4:18 min, 31.6 and 30.0 actions, contact times of 6.4 s and 6.2 s, and reach times of 1.4 s and 1.6 s for women and men, respectively. Olympic combined competitions combine all 3 single disciplines starting with speed followed by bouldering and lead and are characterized by high competition loads, long durations of almost 3 h, and relatively short resting periods in between.

## Introduction

International climbing competitions were first held in 1989 and since then, the research interest of sport and exercise scientists has steadily increased. One of their main concerns is to optimize athlete performance by, for instance, developing performance structure models. Sport-specific requirements, which underlie these models, are derived from competition load structures. In terms of direct applications, this knowledge enables researchers to develop specific testing protocols which imitate competition load structure and permit the design of sport-specific training and conditioning programs that enhance the effectiveness of training for sports performance (Rhea et al., 2006).

Competition load structure can be characterized by internal (individual psychological and physiological response) and external (general characteristics of the competition) measured parameters. Regarding the first, there are numerous studies in sport climbing, which have examined the load structure in simulated competition situations based on such parameters as VO_2_ max (Billat et al., 1995; Mermier and Robergs, 1997; Watts et al., 2000; Sheel et al., 2003; Bertuzzi et al., 2007), lactate concentration (Bertuzzi et al., 2007; Gajewski et al., 2009; Gáspari et al., 2015), and heart rate (Billat et al., 1995; Watts et al., 2000; Sheel et al., 2003; Gajewski et al., 2009; Fuss et al., 2020). External parameters can, in contrast to the internal ones, be assessed free of repercussion on the athlete's performance and obtained from real competitions. This feature notwithstanding, studies investigating the load structure based on external parameters are uncommon in comparison to those that investigate internal parameters.

Today's present competition climbing disciplines are speed, lead, bouldering, and Olympic combined (International Federation of Sport Climbing, 2020).

No studies exist regarding the load structures of speed or Olympic combined climbing competitions. For bouldering, White and Olsen (White and Olsen, 2010) analyzed the load structure of a national bouldering competition in the United Kingdom in 2010. Six elite-level male climbers were analyzed on two selected boulder problems. The structure of the competition was similar to the one nowadays used in the qualification and semi-final rounds of the bouldering World Cups with alternating climbing and resting times of equal durations. While climbing, athletes attempted the boulder problems on average 2.8 times with one attempt lasting 29.8 s. Before starting the initial attempt, athletes spent an average of 75.3 s viewing the boulders and rested 114.5 s between attempts. This meant a 1:3.8 exercise to recovery ratio between climbing and resting intervals. During attempts, the athletes spent more time in dynamic than in static positions and had longer hand contact time than reach times (7.9 s vs. 0.6 s). A similar approach was taken by Medernach et al. (2016), who analyzed the 20 best competitors of each gender on 3 selected boulder problems during the qualification round of a bouldering World Cup in 2013. Women did on average 5.1 attempts per boulder with a duration of 15.2 s and a resting time between attempts of 33.4 s whereas men did 4.3 attempts with a duration of 23.8 s and resting time between attempts of 27.2 s. Another approach was taken by Augste et al. (2021), who classified the boulder problems in so-called boulder types according to their predominant characteristics and analyzed their frequencies and the athlete's success rates. Dynamic moves occurred most frequently and represented the types with which athletes most struggled. However, the studies from White and Olsen (2010) and Medernach et al. (2016) are either limited to male athletes (White and Olsen, 2010), to certain modus (White and Olsen, 2010; Medernach et al., 2016), or to a relatively small number of analyzed climbing performances (White and Olsen, 2010; Medernach et al., 2016). Furthermore, as is to be expected, the evolution of the sport and changes in rules, such as time allowance per boulder, and route setting styles result in a different load structure than back in 2010 or 2013. For lead, Schädle-Schardt (1998) determined the load structure during three national or international championships between 1989 and 1993. Twenty-three athletes (11 women and 12 men) were studied during the final round of each competition. The single attempts consisted of an average of 36.4 moves for women and 42.3 moves for men. This corresponds to an average total climbing time of 4.04 min for women and 4.25 min for men, respectively. The average overall hand contact time was 9.0 s and the average total reach time was 2.4 s with no significant differences between subgroups. Years later, Arbulu et al. (2015) studied 8 women and 8 men at the lead final of the World Climbing Championships in 2012. The total climbing time was almost 6 min for women and less than 4 min for men. Significant differences between women and men were found in frequency and the duration of hand contact, chalking, and resting. For example, the hand contact times were 8.5 s for women and 7.0 s for men. However, in international lead climbing competitions, various rule changes and changes in climbing and route setting styles have occurred in recent years, meaning that the cited previous findings are no longer necessarily valid. Furthermore, the flash modus, which is applied nowadays during the qualification round, has not yet been studied (Schädle-Schardt, 1998; Arbulu et al., 2015).

To summarize, representative values of external measured parameters from current competitive climbing on an international level are still missing. The aim of our study was therefore to analyze the external load structure to determine the general characteristics of competitive climbing at an international level in today's present competition climbing disciplines.

## Materials and Methods

### Procedure

For the analysis of the discipline-specific load structure, video recordings of international climbing competitions were used. For bouldering and lead, we selected a 2018 World Cup and the 2018 World Championship. For speed, one 2018 World Cup was analyzed; for the Olympic combined discipline, the 2018 World Championship was selected. The discrepancies regarding the number of analyzed competitions are rooted in the standardization of the route in speed climbing and the rarity of Olympic combined as an international competition format. The analyzed videos were either obtained from the International Federation of Sport Climbing (IFSC) YouTube channel or were the respective competition's own recordings (Casio EXILIM EX-F1 Cameras, sample rate 30 Hz, speed: 300 Hz). Videos were analyzed using Kinovea (Version 0.8.15) software.

### Variables

The variables analyzed were those considered relevant to describing the load structure in each respective discipline.

Speed: Speed climbing is carried out as a race of two competitors belayed by auto belay systems on two identical, standardized routes. The load structure of each run is characterized by the *start time*, the *number of actions, contact times*, and *reach times*. The *number of actions, contact times*, and *reach times* were assessed for upper and lower limbs independently. The *start time* was calculated as the time difference between the starting signal and the visible beginning of the motor action of the hips in the rightward direction. Athletes have to start at the end of the acoustic countdown which can be anticipated (International Federation of Sport Climbing, 2018). If the athlete reacts to the start signal in less than 0.01s, this is considered as a false start. However, the measurement device is a so-called starting pad for one foot, which means that it is possible for motor action of the hips to commence before the start signal (negative values) without being considered as false start as long as the foot maintains contact with the starting pad for the required time. Due to the high temporal resolution of the camera (300 Hz), it was possible to accurately capture the beginning of the movement. For the purpose of measuring the *number of actions*, an action was determined to be a visible displacement of the limb across the phase of the loss and regaining of contact between holds. *Contact times* were calculated as the time span between the first contact with the climbing holds or the climbing wall and its complete loss of contact. *Reach times*, meanwhile, were calculated as the difference between the loss of contact and the start of the next contact.

Bouldering: In bouldering, short climbs (boulders) at jumping height were protected by landing mats have to be climbed in as few attempts as possible. Bouldering competitions consist of 3 rounds with different modes. In the qualification and semi-final round, a course of boulders has to be climbed in the prescribed order within a fixed time period of 5 min for each boulder, which equals the resting time between boulders. In contrast, in the final round, each boulder is attempted by all competitors before they move on to the next boulder as a group. The climbing time is limited to 4 min. A collective observation period of 2 min per boulder precedes the final but not the qualification or semi-final round. Because the qualification and semi-final round share the same mode, they were considered together and contrasted with the final round. To determine the load structure, the following parameters were quantified in accordance with the IFSC Rules 2018 (International Federation of Sport Climbing, 2018): the *number of attempts per boulder, observation time* as the time between the start of the climbing period and the beginning of the first attempt, *attempt duration* differentiated between successful and not successful attempts and average attempt duration, *climbing time per boulder* as the sum of attempt durations per boulder, *resting time between attempts* and *resting time between boulders, resting time per boulder* as the observation time plus the sum of resting times between attempts, and the *ratio of climbing and resting time per boulder*.

Lead: In lead climbing, the competitors attempt routes on walls a minimum of 15 m high while having to clip the rope into protection points for safety reasons. Progression up the wall is the main scoring criteria. Two different modes are applied during lead climbing competitions, namely, “flash” (used in the qualification round) and “onsight” (used in the semi-final and final round). These modes differ in the amount of route information available to the athletes before they climb. Accordingly, the qualification round was contrasted with the semi-final and final rounds, which were considered together due to sharing the same mode. To determine the load structure, *contact times* and *reach times*, the *number of actions*, and the *total climbing time* were analyzed. *Contact times* and *reach times*, and the *number of actions* were measured in the same way as in speed climbing. Additionally, bodyside and finger grip position (open hand grip vs. crimp grip) were noted. The *reach times* of the upper limbs were further subdivided according to whether the athletes were chalking to reduce sweat, clipping the rope into the protection points, shaking their arms for recovering, or just aiming to grab the next hold (“grabbing only”). Combinations of these movements were also considered. *Total climbing time* was measured according to the IFSC Rules (International Federation of Sport Climbing, 2018) as the time span between the start of the attempt and the moment where either the final quickdraw of the route was clipped or a fall occurred and the contact with all extremities to the holds or the climbing wall was lost.

Olympic combined: In the Olympic combined event, athletes compete against each other in the abovementioned single disciplines. During the final round, athletes compete in all three single disciplines within one competition, starting with speed, bouldering, and then lead. In speed, the athletes have to do either 1 or 3 runs depending on whether they advance to the next stage or not. Both, the bouldering and lead parts follow the final round format of standard competitions in the disciplines described above. Observation time in each discipline takes place during the resting time between events. The final ranking is determined based on multiplying the athlete's result in each respective discipline. Where load structure is concerned, the analysis of the overall duration of the competition and the resting times between the single disciplines was prioritized because it was assumed that the load structure of the single disciplines is similar to the load structure of the single disciplines carried out within the Olympic combined competition. The *overall duration of the competition* was measured as the time span between the beginning of the first attempt of the first speed run and the end of the lead attempt. The *resting time between runs in speed* was measured as the time span between the end and the start of the consecutive speed run, the *resting time between speed and bouldering* as the time span between the end of the last speed attempt and the beginning of the first climbing period in bouldering and, lastly, the *resting time between bouldering and lead*, as the end of the last attempt in bouldering and the beginning of the lead attempt. Further details are described in the respective section of the single disciplines.

### Sample

Subjects were elite athletes competing at international climbing competitions in 2018 and represent the best climbers of the respective competition:

Speed: The 20 fastest runs with available video recordings were analyzed while considering only the 2 fastest runs per athlete. In cases where an athlete completed more than 2 of the 20 fastest runs, runs from the next fastest athlete were selected to enhance variability. The selected runs were scored from 12 women and 14 men athletes, respectively. The runs were analyzed regardless of the round of competition.

Bouldering: Four competition rounds, the qualification, semi-final, and final round from the World Cup and the final round of the World Championship, were included. In both, the qualification and semi-final round, the courses of the semi-finalists (each round: 20 per gender), and in the final rounds, the courses of the finalists (each final round: 6 per gender) were analyzed. As some athletes participated in the selected rounds of both competitions, video recordings of 20 different female athletes and 24 different male athletes were analyzed.

Lead: In total, 37 attempts from female and 43 attempts from male athletes were analyzed. These data represent 8 attempts per gender in both the semi-final and final rounds of the World Cup (16 attempts in total per gender), 6 attempts per gender from the finals of the World Championship, and those 10 attempts by women and 16 attempts by men in which the route was topped in the qualification round. Just as in bouldering, some athletes participated in both competitions, which means that the attempts were obtained from 12 different female and 25 different male athletes.

Olympic combined: Only the final round of the World Championship was analyzed as the qualification round is already covered by studies of the single disciplines, which means that the load structure was obtained from the 6 female and the 6 male finalists.

### Reliability

Two independent raters assessed inter-rater reliability on 5% of the data. The intraclass correlation coefficient (ICC) according to the schema developed by Koo and Li (2016) was used as the reliability coefficient and calculated for each analyzed variable separately. In the case of disagreements regarding the consideration of movements as separate actions where contact and reach times were concerned, data were compared up until the point of disagreement respectively from thereon. Inter-rater reliability of all variables is presented in Table 1.

**Table 1:** Inter-rater reliability of the analyzed dependent variables used for load structure determination.

	**Variables**	**ICC**	**95% CI LL**	**95% CI UL**
Speed	Start time	0.90^*^	−0.01	1.00
	Number of actions upper limbs	1.00^***^	1.00	1.00
	Number of actions lower limbs	1.00^***^	1.00	1.00
	Contact time upper limbs	0.98^***^	0.88	0.99
	Contact time lower limbs	0.93^***^	0.72	0.97
	Reach time upper limbs	0.89^***^	0.70	0.95
	Reach time lower limbs	0.92^***^	0.66	0.97
Bouldering	Number of attempts per boulder	1.00^***^	1.00	1.00
	Successful attempt duration	1.00^***^	1.00	1.00
	Non-successful attempt duration	1.00^***^	1.00	1.00
	Climbing time per boulder	1.00^***^	1.00	1.00
	Average attempt duration per boulder	1.00^***^	1.00	1.00
	Observation time	0.99^***^	0.91	1.00
	Resting time between attempts	1.00^***^	1.00	1.00
	Resting time per boulder	1.00^***^	0.99	1.00
	Resting time between boulders	1.00^***^	1.00	1.00
	Ratio between climbing and resting time	1.00^***^	0.99	1.00
Lead	Total climbing time	0.99^***^	0.92	1.00
	Number of actions upper limbs	0.97^***^	0.85	0.99
	Number of actions lower limbs	0.38	−1.00	0.86
	Contact time upper limbs	0.99^***^	0.99	1.00
	Contact time lower limbs	1.00^***^	1.00	1.00
	Reach time upper limbs	0.99^***^	0.99	1.00
	Reach time upper limbs grapping only	0.97^***^	0.96	0.98
	Reach time lower limbs	0.98^***^	0.98	0.97
Olympic combined	Resting times between runs in speed	1.00^***^	1.00	1.00
	Resting time between speed and bouldering	1.00^***^	1.00	1.00
	Resting time between bouldering and lead	1.00^**^	1.00	1.00
	Total competition duration	1.00^***^	1.00	1.00

* *p < 0.05*,

** *p < 0.01*,

*** *p <0.001*.

The inter-rater reliability was very high across all disciplines for almost all of the analyzed parameters. This conclusion can be drawn from the fact that the relative reliability coefficients (ICC) exceeded the 0.81 benchmark in almost all cases, which according to Hopkins (Hopkins, 2000) indicates high reliability. Exceptions occurred in lead climbing only, where the ICC for the number of actions of the lower extremities was 0.38. This may be due to the fact that the two raters disagreed as to whether short contacts with the wall are a sufficient criterion to rate them as separate actions. However, the difference was rather small (*M* = 1.25, standard deviation [SD] = 2.12) and the ICC could be largely influenced by the small number of observations (*n* = 8).

A general high reliability is in line with existing studies in this field (Schädle-Schardt, 1998; White and Olsen, 2010; Arbulu et al., 2015). This further confirms time-motion analysis as a reliable tool to analyze kinematic parameters in the context of load structure determination.

### Statistical Analyses

IBM® SPSS® Statistics (RRID:SCR_019096, Version 26) was used for all statistical analyses.

For speed, the climbing route is highly standardized, and therefore statistical tests for analyzing group differences were applied: *t-*tests to calculate group differences between genders for the assessed independent variables start time, number of actions upper and lower limbs, and contact and reach times for upper and lower limbs. Statistical significance was accepted at *p* < 0.05 level. For bouldering, lead, and Olympic combined, in contrast, only descriptive data are provided. Due to the dependency of the load structure on route characteristics and climbing style of the athletes, which differ between rounds and genders, no inferential statistics were calculated.

## Results

### Speed

The load structure in speed climbing is determined by two attempts in the qualification round and one attempt in each stage of the final round. Each attempt is characterized by the start time, the number of actions, and the contact and the reach times of the upper and lower limbs. Statistics from those variables are presented in Table 2.

**Table 2:** Statistics of international speed climbing competitions.

		**Start time**	**Number of actions upper limbs**	**Number of actions lower limbs**	**Contact time upper limbs**	**Contact time lower limbs**	**Reach time upper limbs**	**Reach time lower limbs**
Women	*N*	20	40	40	421	427	439	466
	*M*	0.10 s	11.2	11.7	0.25 s	0.39 s	0.49 s	0.33 s
	*SD*	0.08 s	0.5	1.1	0.08 s	0.14 s	13.24 s	11.03 s
	*Median*	0.07 s	11.0	12.0	0.23 s	0.40 s	0.36 s	0.28 s
Men	*N*	20	40	40	365	366	385	406
	*M*	−0.08 s	9.7	10.2	0.24 s	0.36 s	0.38 s	0.23 s
	*SD*	0.09 s	0.8	1.0	0.08 s	0.11 s	0.11 s	0.10 s
	*Median*	−0.07 s	9.0	10.0	0.19 s	0.32 s	0.35 s	0.33 s
Women vs. men	*T*	5.977	10.505	6.471	2.283	3.179	11.712	11.982
	*P*	<0.001	<0.001	<0.001	0.023	0.002	<0.001	<0.001
	*Mean difference*	0.18	1.5	1.6	0.01	0.03	0.12	0.10
	*95% CI LL*	0.12	1.2	1.1	0.00	0.01	0.10	0.08
	*95% CI UL*	0.24	1.8	2.0	0.02	0.05	0.14	0.11

*Sample: 20 runs from 12 female and 14 male athletes, respectively. N, sample size; M, mean; SD, standard deviation; T, t-value; P, p-value; CI LL, confidence interval, lower limit; CI UL, confidence interval, upper limit*.

Group comparison showed significant differences between the load characteristics of women and men for all of the analyzed parameters. Consistently, longer durations were found in the women's than in the men's category.

### Bouldering

The structure of bouldering competitions consists of a course of boulders (5 in the qualification round and 4 in the semi-final and final round) which have to be climbed within a fixed time period. Within each climbing period, the athletes attempted a boulder problem 3 times in the qualification-/semi-final round and 4 times in the final round at a median with an average attempt duration of 27.0 s and an average resting time between attempts of 32.2 s. Due to the different modes applied, the resting time between boulders was around 8 min in the qualification-/semi-final round and 22 min in the final round.

Details about the load structure in bouldering are presented in Table 3.

**Table 3:** Statistics of international bouldering competitions.

		**Number of attempts per boulder**	**Successful attempt duration**	**Non-successful attempt duration**	**Climbing time per boulder**	**Average attempt duration per boulder**	**Observation time**	**Resting time between attempts**	**Resting time per boulder**	**Resting time between boulders**	**Ratio between climbing and resting time**
Qualification and semifinal round women	*N*	177	91	485	176	173	175	397	173	70	173
	*M*	3.2	39 s	21 s	76 s	28 s	47 s	35 s	125 s	498 s	0.72
	*SD*	2.0	14 s	14 s	42 s	13 s	11 s	21 s	66 s	167 s	0.42
	*Median*	3	36 s	18 s	68 s	25 s	48 s	33 s	119 s	421 s	0.60
Qualification and semifinal round men	*N*	180	111	548	175	175	172	420	135	67	134
	*M*	3.4	32 s	13 s	62 s	27 s	51 s	34 s	113 s	430 s	0.68
	*SD*	2.7	13 s	13 s	33 s	18 s	13 s	22 s	73 s	84 s	0.49
	*Median*	3	29 s	8 s	54 s	24 s	50 s	31 s	90 s	394 s	0.56
Final round women	*N*	48	25	202	48	48	48	173	47	36	47
	*M*	4.4	39 s	12 s	70 s	27 s	16 s	22 s	96 s	1308 s	1.72
	*SD*	4.0	19 s	14 s	34 s	24 s	8 s	14 s	68 s	366 s	2.39
	*Median*	4	35 s	6 s	70 s	24 s	15 s	19 s	91 s	1468 s	0.94
Final round men	*N*	52	26	182	52	52	52	142	52	39	52
	*M*	3.7	31 s	16 s	73 s	24 s	20 s	32 s	109 s	1416 s	1.10
	*SD*	1.8	12 s	13 s	33 s	15 s	10 s	21 s	57 s	165 s	1.18
	*Median*	4	28 s	12 s	66 s	21 s	19 s	28 s	112 s	1419 s	0.69

*Sample: 52 courses from 20 female and 24 male athletes, respectively. N, sample size; M, mean; SD, standard deviation*.

### Lead

The load structure in lead climbing is characterized by two attempts in the qualification round with a minimum resting time of 50 min in-between and by a single attempt in the semi-final and final rounds. Every attempt is characterized by an average climbing time of 4:09 and 4:18 min, 31.6 and 30.0 actions, contact times of 6.4 and 6.2 s, and reach times of 1.4 and 1.6 s in the women's and men's category, respectively. Statistics analyzing the climbing, contact and reach times, and the number of actions in a more detailed way are presented in Table 4.

**Table 4:** Statistics of climbing, contact and reach time, and the number of actions at international lead climbing competitions.

		**Total climbing time**	**Number of actions upper limbs**	**Number of actions lower limbs**	**Contact time upper limbs**	**Contact time lower limbs**	**Reach time upper limbs**	**Reach time upper limbs grapping only**	**Reach time lower limbs**
Qualification round women	*N*	10	20	20	584	705	564	275	682
	*M*	3:39 min:s	29.4	35.5	8.01 s	6.98 s	2.17 s	0.69 s	1.21 s
	*SD*	0:48 min:s	4.8	5.2	4.81 s	7.04 s	2.23 s	1.14 s	1.56 s
	*Median*	3:40 min:s	28.5	35.5	7.60 s	4.87 s	1.36 s	0.43 s	0.73 s
Qualification round men	*N*	16	32	32	874	1,000	831	473	958
	*M*	4:55 min:s	27.3	31.3	6.61 s	6.18 s	2.07 s	0.65 s	1.38 s
	*SD*	0:49 min:s	3.5	4.2	3.64 s	8.05 s	2.21 s	0.71 s	1.94 s
	*Median*	4:40 min:s	27.5	32.0	6.10 s	3.87 s	0.77 s	0.47 s	0.77 s
Semifinal and final round women	*N*	26	52	52	1,393	1,602	1,356	728	1,587
	*M*	4:21 min:s	27.9	34.6	5.63 s	6.13 s	1.50 s	0.62 s	1.20 s
	*SD*	0:56 min:s	7.2	4.2	4.95 s	6.32 s	2.49 s	2.39 s	1.57 s
	*Median*	4:11 min:s	27.5	34.5	5.25 s	4.17 s	0.53 s	0.43 s	0.8 s
Semifinal and final round men	*N*	26	52	52	1,432	1,535	1,398	792	1,519
	*M*	3:55 min:s	28.8	32	6.30 s	5.98 s	1.86 s	0.69 s	1.34 s
	*SD*	1:08 min:s	8.1	9.2	4.04 s	7.43 s	1.97 s	1.00 s	2.13 s
	*Median*	3:52 min:s	28.0	30.5	5.95 s	3.73 s	0.77 s	0.47 s	0.73 s

*Sample: 37 attempts from 12 female athletes and 43 attempts from 25 male athletes. N, sample size; M, mean; SD, standard deviation*.

The average durations of contact and reach times were equal between the left and the right bodyside for both the upper and lower limbs. Reach times of the upper limbs were further subdivided: reaching directly (grabbing only) for the next hold occurred most frequently (54.9%) and lasted up to 1 s in 90.8% of the cases. In 81.9% of cases, reaching directly for the next hold occurred either alone or in combination with the other categories. Athletes shook their hand, clipped, or chalked in 4.0, 3.4, and 2.3% of the actions, though when combined with grabbing, these percentages were increased to 4.3, 4.4, and 8.1%, respectively. Shaking alone or in combination with other categories (“shaking any”) were occurred in 24.5% of the actions, “clipping any” in 21.3% and “chalking any” in 19.5%. For those, a different frequency distribution pattern of the durations had been found in comparison to “grabbing only” with the majority of the reach times lasting longer than 3 s for “shaking any,” longer than 2 s for “clipping any,” and longer than 5 s for “chalking any.”

Regarding finger grip positions: In the women's category, the crimp grip was applied in 66.5% of the cases and the open hand grip in 33.5% whereas, in the men's category, the crimp and open hand crimp were applied in 36.7 and 63.3% of the cases, respectively.

### Olympic Combined

The load structure of the final round of Olympic combined is considered apart from that of the single disciplines and is characterized by the resting time between the runs in speed, the resting time between speed and bouldering and between bouldering and lead, and the total duration of the competition. Statistics are presented in Table 5.

**Table 5:** Statistics of international Olympic combined climbing competitions.

		**Resting times between runs in speed**	**Resting time between speed and bouldering**	**Resting time between bouldering and lead**	**Total competition duration**
Final round women	*N*	8	6	6	6
	*M*	6:41 min:s	31:11 min:s	41:55 min:s	2:52 min:s
	*SD*	1:20 min:s	4:32 min:s	9:17 min:s	0:12 min:s
	*Min*	4:14 min:s	24:52 min:s	35:10 min:s	2:35 min:s
	*Max*	8:34 min:s	37:22 min:s	59:07 min:s	3:07 min:s
Final round men	*N*	8	6	6	6
	*M*	6:08 min:s	30:41 min:s	34:31 min:s	2:29 min:s
	*SD*	1:41 min:s	3:21 min:s	8:47 min:s	0:11 min:s
	*Min*	3:19 min:s	26:28 min:s	24:02 min:s	2:14 min:s
	*Max*	8:20 min:s	36:26 min:s	45:43 min:s	2:44 min:s

*Sample: 6 competition courses from 6 athletes per category. N, sample size; M, mean; SD, standard deviation; Min, minimum; Max, maximum*.

## Discussion

### Speed

The median number of actions was varied between 9 and 12 for the upper and the lower limbs, respectively, with men carrying out fewer actions than women. These gender-related differences are caused by the fact that men tend to skip hold 7 referring to numbering all holds that include footholds in an ascending order from the ground. This is well known as the so-called “Tomoa Skip.” Higher up, women used hold 18 with their right hand whereas men directly went from hold 14 to 19 skipping hold 18. However, very low SDs show that the movement sequences of the top athletes are very standardized.

Movement speed can be derived from the sum of contact and reach times resulting in an average movement time for the upper and lower limbs of 0.74 and 0.72 s in the women's and 0.62 and 0.59 s for the men's category, respectively. Not considering the differentiation between the upper and lower limbs, the average movement time was 0.73 s in the women's and 0.60 s in the men's category. Men carried out fewer actions than women and therefore had to cover a greater distance per action and nonetheless also had significantly shorter contact and reach times. However, this is hardly surprising given that men and women compete on an identical route despite having different constitutions.

Notably, contact times recorded are shorter than those found by Fuss and Niegl (2006). On the one hand, this could be due to the fact that their study was conducted on a non-standardized route; on the other, it could be due to the fact that athletes have become significantly faster in recent years. For example, between 2009 and 2018, the fastest time at the World Championships was improved from 9.3 to 7.65 s for women and from 6.64 to 5.63 s for men (International Federation of Sport Climbing, 2021). The differences between women and men might be related to sex-related strength differences (Sandbakk et al., 2018).

Another interesting finding is that the starting time was on average 0.01 s in the women's and −0.08 s in the men's category. Due to the fact that the start signal can be anticipated (International Federation of Sport Climbing, 2020), athletes partly started the movement before the last beep of the countdown. Such short or even negative starting times seem likely with years of practice taking into account the findings from Borysiuk and Sadowski (2007), who observed a significant reduction in latent reaction time due to time anticipation within a single experiment. The ability to precisely anticipate and appropriately coordinate the movement start may imply a high-performance benefit.

In terms of practical application, the recommended number of repetitions for sport-specific testing and training protocols should reflect the median number of actions, which varies from 9 and 12. Furthermore, the movement speed of around 0.73 and 0.6 s for women and men, respectively, serves as an additional training parameter and biometric feedback tool for training control (Weakley et al., 2021). Additionally and as already mentioned, minimizing starting times holds the potential to significantly reduce speed running times.

### Bouldering

The load structure in bouldering is characterized by bouts of activity punctuated with resting periods. Therefore, the ability to recover seems crucial. Well-trained recovery ability enables competitors to shorten the resting times between attempts, therefore, permitting them to make more attempts within each climbing period and through the course of the entire competition. The importance of endurance for bouldering performance is supported by the findings of numerous studies (Fryer et al., 2017; Michailov et al., 2018; Stien et al., 2019). Other factors contributing to success in bouldering are high bouldering skills and excellent onsight/flash climbing abilities. Furthermore, another key component was found to be the discovery of new creative solutions after an unsuccessful attempt (Künzell et al., 2021).

The current study updates and expands existing knowledge due to its relatively large sample size and its analysis of all rounds of current competition bouldering when compared to the study of a national competition by White and Olsen (2010) and to the study of World Cups 5 years earlier by Medernach et al. (2016). The comparison of concrete results with the study of Medernach et al. (2016), who analyzed a competition that was held in the same format, shows that in our study women executed fewer attempts (*M* = 3.2 vs. *M* = 5.1) but with longer durations (*M* = 28 s vs. *M* = 15 s). Men executed fewer attempts as well (*M* = 3.4 vs. *M* = 4.3) and rested longer in between them (*M* = 34 s vs. *M* = 27 s). This indicates a trend toward executing fewer but more well-planned attempts while focusing on recovery between them in order to increase success. This underlines the necessity of up-to-date competition analyses in order to map the load structure correctly and help athletes to be prepared in the best way possible.

In terms of practical application: In bouldering, the load structure is determined by alternating climbing and resting periods. Therefore, in order to simulate the load structure of bouldering competitions, it is recommended to train multiple (Billat et al., 1995; Mermier and Robergs, 1997) high-intensity efforts (equaling the number of attempts) with durations of around 30–40 s (equaling average and successful attempt durations) followed by resting periods of around 20–30 s (equaling the resting time between attempts) and to perform 4–5 sets (equaling the number of boulders) with serial rests of either 8 or 22 min between sets (equaling the resting time between boulders).

As recovery ability is paramount in bouldering, recovery strategies targeting the different resting times found in bouldering competitions should be developed and practiced.

### Lead

The current study provides valuable insights into the current load structure in competitive lead climbing.

As already stated, various rule changes and changes in climbing and route setting styles have occurred in recent years, which could explain the differences between studies. In detail, the trend toward a more dynamic and faster climbing style is reflected in the contact and reach times, which were consistently lower in this study compared to the previous ones [contact time upper limbs women: *M* = 5.63 s vs. *M* = 10.3 s (Schädle-Schardt, 1998) vs. *M* = 8.5 s (Arbulu et al., 2015), contact time upper limbs men: *M* = 5.63 s vs. *M* = 9.1 s (Schädle-Schardt, 1998) vs. *M* = 7.0 s (Arbulu et al., 2015), reach time upper limbs women: *M* = 1.50 s vs. *M* = 2.4 s (Schädle-Schardt, 1998), and reach time upper limbs men: *M* = 1.86 s vs. *M* = 2.5 s (Schädle-Schardt, 1998)].

Since reaching directly for the next hold ("grabbing only”) accounts for only 54.9% of the cases, contact and reach times are highly influenced by the frequencies and durations of shaking, clipping, and chalking of the athletes, which might contribute to the much shorter durations reported above in comparison to the findings of Schädle-Schardt (Schädle-Schardt, 1998) and Arbulu et al. (2015).

Short contact and reach times and high climbing speed might positively influence the climbing economy if the route requirements tend to exceed the athlete's critical force (Giles et al., 2021) and maintaining a stronger pace throughout the attempt has been shown to be beneficial for competition climbing success (Arbulu et al., 2015; Kotchenko, 2017). According to routesetters, women had to face difficulties of around 8b in the qualification round and 8b+/8c in the final rounds whereas men faced difficulties of 8b+/8c in the qualification and 8c+ in the final rounds.

A practical implication, which can be derived from the load structure in lead climbing for performance analysis, for example, is the assessment of climbing-specific intermittent finger endurance. Different test protocols have been used but the ones that are representative of the load structure of current competition climbing should be prioritized (Michailov et al., 2018). Based on the results, this would mean a 6 s work to 2 s rest ratio representing the overall averages of contact and reach times.

### Olympic Combined

The combination of all 3 single disciplines within the final round of Olympic combined climbing competitions results in a higher competition load as compared to the single disciplines.

The average resting time between the runs in speed climbing was 6:24 min (SD = 1:29). Minimal and maximal resting times were 3:19 and 8:34 min, respectively. This relatively large deviation could be explained by the competition format.

The same accounts for the resting times between speed and bouldering and between bouldering and lead. Due to the starting order of the subsequent disciplines being in reverse order with respect to ranking up until this point in the competition, the differences between the minimal and maximal resting times were roughly 13 and 10 min between speed and bouldering and 24 and 21 min between bouldering and lead in the women's and men's category, respectively.

Given the high competition loads and the relatively short resting times between disciplines, a practical application of this study's result may be that athletes should not only train in a way to maximize performance in the single disciplines but to also handle the required high load, long duration, and short resting times of the combined format.

Crucial aspects for targeting this are tailored fueling (Michael et al., 2019) and recovery strategies. The latter must be highly effective and at the same time require a very limited amount of time. The reason for this is that in practice, the resting times can only partially be used for recovery due to fact that the observation period of the following discipline (4 times 2 min for bouldering and 6 min for lead), immediate climbing preparation, and other activities take place during this period.

Evidence indicates that a few minutes of active recovery either by walking or easy climbing (Draper et al., 2006; Valenzuela et al., 2015) lead to improved recovery and therefore are considered a tailored recovery strategy. For cold water immersion, in contrast, only durations of 20 min have been evaluated (Heyman et al., 2009) while the benefit of shorter periods remains unclear.

## Limitations

The main limitation of the current study is the dependency of load structure on route characteristics. These vary greatly from competition to competition and even from boulder/route to boulder/route and therefore it is generally difficult to derive a universally valid load structure. Single influences were reduced by a broader data basis, which was derived from the selection of different routes and courses of competitions from different competitions. Nevertheless, the current study is limited in regard to the chosen sample and by virtue of not considering a wider range of competitions.

Furthermore, load structure is dependent on an athlete's climbing style. By analyzing the world's best climbers in each discipline, the presented load structure tends to be more valid for high-level athletes who compete at international climbing competitions than for lower level climbers who have different climbing abilities and might not be able to use the same methods (e.g., the so-called Tomoa skip in speed climbing). These athletes should therefore use load structures that represent the requirements of their own climbs and climbing abilities.

Another limitation arises from the constant development of climbing and route setting styles, which means that the presented load structure based on 2018 competitions does not necessarily represent the requirements of current competitions. This is especially true of the Olympic combined where a new format will be applied in the 2024 Olympic Games. Generally speaking, a retrospective approach is an inherent problem of this research area.

## Data Availability Statement

The raw data supporting the conclusions of this article will be made available by the authors, without undue reservation.

## Ethics Statement

The studies involving human participants were reviewed and approved by Commitee for Ethics University of Augsburg. Written informed consent from the participants' legal guardian/next of kin was not required to participate in this study in accordance with the national legislation and the institutional requirements.

## Author Contributions

All authors conceived of and designed the analysis, collected the data, discussed the results, and contributed to the final manuscript. MW performed the data and statistical analysis and took lead in writing and editing the manuscript. By providing critical feedback and revising the initial manuscript, CA and SK helped shaped the final manuscript. CA supervised the project. All authors contributed to the article and approved the submitted version.

## Funding

This study was part of the research project Development of a scientifically based performance diagnostics in sports climbing which was founded by the German Federal Institute of Sport Science (BISp ZMVI4-070707/18-19).

## Conflict of Interest

The authors declare that the research was conducted in the absence of any commercial or financial relationships that could be construed as a potential conflict of interest.

## Publisher's Note

All claims expressed in this article are solely those of the authors and do not necessarily represent those of their affiliated organizations, or those of the publisher, the editors and the reviewers. Any product that may be evaluated in this article, or claim that may be made by its manufacturer, is not guaranteed or endorsed by the publisher.
